# Crashworthiness Enhancement of Kelvin-Cell Lattice Structures Through CFRP Rod Reinforcement: An Experimental and Data-Driven Assessment

**DOI:** 10.3390/polym18141686

**Published:** 2026-07-08

**Authors:** Hamdi Kuleyin

**Affiliations:** Department of Mechanical Engineering, Faculty of Engineering and Architecture, Recep Tayyip Erdogan University, 53100 Rize, Türkiye; hamdi.kuleyin@erdogan.edu.tr

**Keywords:** Kelvin-cell lattice structures, polymer lattice structures, CFRP rod reinforcement, crashworthiness, energy absorption, data-driven analysis, mechanical properties

## Abstract

Lattice structures are widely utilized in lightweight engineering due to their design flexibility and tailorable mechanical properties. However, polymer lattices often exhibit limited load-bearing capacity and moderate crashworthiness under compression. This study proposes a hybrid reinforcement strategy based on the integration of carbon fiber-reinforced polymer (CFRP) rods into polymeric Kelvin-cell lattices. The specimens were manufactured via masked stereolithography, and the effects of rod placement pattern, the number of rods, and rod-length configuration were systematically investigated under quasi-static compression. Crashworthiness was evaluated in terms of force–displacement response, deformation mode, and crashworthiness metrics. Compared with the empty Kelvin-cell lattice, the best-performing hybrid configuration increased energy absorption, specific energy absorption, and mean crushing force by approximately 356%, 307%, and 356%, respectively. Mechanistically, distributed rod placement promoted more uniform load sharing, while the effect of increasing rod number depended strongly on the rod-length configuration. In addition, delayed or sequential reinforcement strategies provided superior performance and an enhanced balance between energy absorption and force efficiency. Regression models and ANOVA consistently identified rod-length configuration as the dominant design variable. These findings demonstrate that CFRP rod reinforcement can effectively enhance the crashworthiness of polymeric Kelvin-cell lattices, provided that the rod placement pattern, rod number, and rod-length configuration are designed jointly.

## 1. Introduction

Lightweight lattice structures have garnered significant attention across various engineering sectors owing to their high specific stiffness and strength, tailorable deformation behavior, multifunctional design capability, and superior energy absorption potential. These architectures are particularly suitable for structural components where weight reduction, mechanical performance, and crashworthiness must be realized simultaneously. Advances in additive manufacturing have further expanded the application envelope of lattice structures by enabling the fabrication of complex geometries with high dimensional accuracy and design flexibility [1]. Among various lattice topologies, the Kelvin-cell lattice, characterized by its tetrakaidekahedral geometry, is recognized as an efficient space-filling architecture with relatively isotropic mechanical characteristics [2]. Due to its uniform three-dimensional partitioning and minimized surface area, the Kelvin-cell topology offers stable deformation behavior, compressive stiffness, and shear resistance, rendering it highly advantageous for lightweight and energy-absorbing applications [3,4,5].

The mechanical response of Kelvin-cell lattices is strongly governed by architectural parameters, including strut cross-sectional shape, strut diameter, strut length, fillet radius, cell size, and relative density [6,7,8,9]. Consequently, their stiffness, plateau behavior, deformation mode, and energy absorption capacity can be tailored by modifying both the unit-cell geometry and the overall lattice architecture. Recent studies have also demonstrated the potential of Kelvin-cell lattices for impact mitigation and personal protective equipment. For example, Trottier et al. demonstrated that elastomeric Kelvin-cell lattices can provide higher specific energy absorption, reduced impact acceleration, and a more stable plateau response compared with their body-centered cubic (BCC) and face-centered cubic (FCC) counterparts [10]. Similarly, Xu et al. reported that Kelvin-cell-based and foam-filled graded hybrid structures offer multifunctional energy-absorption capabilities for helmet applications under impact loading [11]. Despite these advantages, polymeric Kelvin-cell lattices nonetheless exhibit limited load-bearing capacity and moderate crashworthiness under demanding compressive or impact conditions.

To overcome these limitations, several enhancement strategies have been investigated, including geometric grading, modification of strut geometry, relative-density optimization, and the hybridization of Kelvin-cell-based structures. Montgomery et al. examined functionally graded Kelvin-cell structures produced via digital light processing (DLP) and reported that spatial variations in the degree of curing improved energy absorption compared to uniform structures [12]. Similarly, studies on Kelvin-cell lattices manufactured by fused filament fabrication have shown that infill density and relative density profoundly influence the mechanical response, deformation mode, and energy absorption under quasi-static and dynamic loading [13]. Ge et al. scrutinized thermoplastic polyurethane Kelvin-cell structures and reported promising cyclic compression, cushioning, and energy absorption behaviors [14]. Horizontally and vertically graded Kelvin-cell structures have also been shown to provide stable impact responses and enhanced energy absorption capabilities [15,16]. These studies indicate that the crashworthiness of Kelvin-cell lattices is highly design-dependent; nonetheless, additional strategies are required to substantially elevate their load-bearing and energy-absorption capacities without compromising lightness.

Against this backdrop, hybrid structural design concepts and fiber-reinforced supporting elements represent promising approaches for enhancing the mechanical performance of lattice and cellular structures [17,18]. Carbon fiber-reinforced polymer (CFRP) components are particularly advantageous due to their high specific stiffness and strength. CFRP rods consist mainly of continuous fibers aligned along the longitudinal direction, offering low density, high axial stiffness and strength, corrosion and fatigue resistance, and consistent geometry. These features enable their use as prestressing tendons, cable elements, strengthening members, and lightweight load-bearing components in various structural applications [19,20]. However, their anisotropic nature makes their compressive behavior sensitive to slenderness, alignment, lateral support, and local damage. Therefore, both the unsupported length and surrounding structural support significantly affect their load-carrying performance under compression. Previous studies on carbon fiber-reinforced lattice and truss structures have revealed that fiber-reinforced members substantially enhance load-carrying capacity and specific mechanical performance along the fiber direction [21,22]. Continuous carbon fiber-reinforced lightweight lattice structures have also been investigated as sandwich panel cores and have demonstrated competitive specific mechanical properties compared to metallic and ceramic lattices at similar relative densities [23,24]. However, the direct fabrication of continuous unidirectional fiber-reinforced struts within complex lattice topologies such as Kelvin-cell structures remains highly challenging [25]. In addition, carbon fiber-reinforced struts are prone to sudden failure and an abrupt loss of load-carrying capacity during deformation [26]. Consequently, instead of replacing the lattice struts themselves, strategically inserting fiber-reinforced elements into selected cell cavities offers a novel and viable reinforcement strategy.

The effectiveness of inserted fiber-reinforced elements has been widely validated across various cellular and sandwich structures. For instance, carbon fiber tubes inserted into aluminum honeycomb cores significantly enhance compressive behavior, impact resistance, and energy absorption capacity [27,28,29,30]. These findings suggest that the strategic placement of fiber-reinforced elements within cellular architectures can substantially optimize crashworthiness. Moreover, recent studies on continuous carbon fiber-reinforced and hybrid composite lattice systems have indicated that reinforcement arrangement, fiber orientation, and hybridization strategies strongly influence stiffness, strength, deformation mode, and energy absorption behavior [31,32]. Therefore, the mechanical efficiency of reinforced lattice structures is governed not only by the reinforcement material itself but also by its architectural distribution.

Although previous studies have demonstrated the potential of Kelvin-cell lattices, functionally graded cellular structures, and carbon-fiber-reinforced lattices, the role of externally inserted CFRP rods in polymeric Kelvin-cell lattices remains to be systematically clarified. In particular, the combined effects of the rod placement pattern, number of rods, and rod-length configuration on the crushing response, deformation mechanisms, and energy absorption remain insufficiently understood. To address this gap, this study experimentally evaluates the crashworthiness behavior of CFRP rod-reinforced Kelvin-cell lattices under quasi-static compression, while supporting the interpretation of the experimental findings through a data-driven assessment. Polymeric Kelvin-cell lattices were fabricated via masked stereolithography (MSLA) and reinforced with CFRP rods arranged according to different placement patterns, rod numbers, and rod-length configurations. The effects of these design variables on force–displacement response, deformation behavior, energy absorption, specific energy absorption, peak crushing force, initial peak crushing force, mean crushing force, and crushing force efficiency were systematically investigated. Unlike previous studies focusing mainly on unit-cell topology, relative density, material grading, or continuous fiber path design, the present work clarifies how discrete CFRP rods modify the mechanical response and crashworthiness of polymeric Kelvin-cell lattice structures. Furthermore, data-driven analyses were employed to quantify the relative influence of these design factors and support the interpretation of their interactions.

## 2. Materials and Methods

This section presents the experimental and data-driven methodology employed to evaluate CFRP rod-reinforced Kelvin-cell lattices. The subsequent subsections describe the lattice design and fabrication, reinforcement variables, mechanical testing procedures, crashworthiness metrics, regression modeling, and statistical analysis.

**Figure 1:**
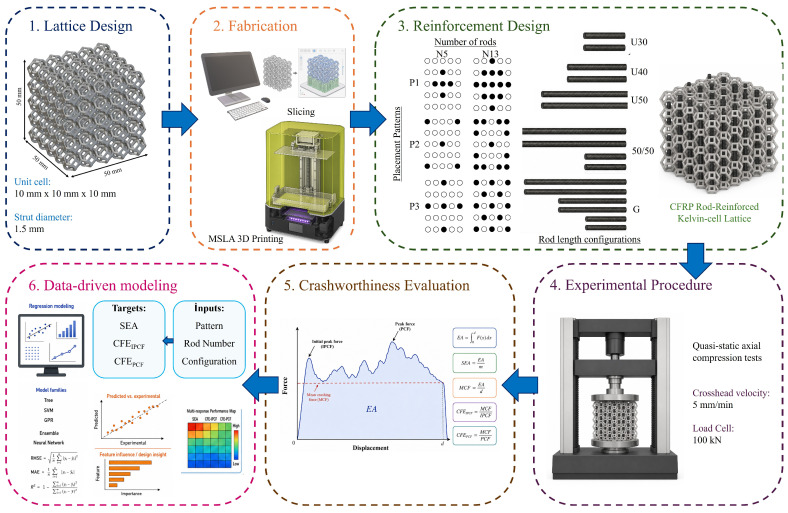
Methodological workflow for the experimental and data-driven assessment of CFRP rod-reinforced Kelvin-cell lattice structures.

Figure 1 illustrates the methodological framework adopted in this study. The workflow begins with the geometric design of the Kelvin-cell specimens, followed by the fabrication of the polymeric lattices. The reinforcement stage incorporates the principal design variables: the CFRP rod placement pattern, number of rods, and rod-length configuration. The reinforced specimens were then subjected to quasi-static axial compression, and the resulting force–displacement responses were used to calculate the crashworthiness metrics. Finally, regression-based machine learning models were developed from the experimental dataset to assess the influence of the design variables on the crashworthiness of the CFRP rod-reinforced Kelvin-cell specimens.

### 2.1. Design and Fabrication of Kelvin-Cell Lattices

**Figure 2:**
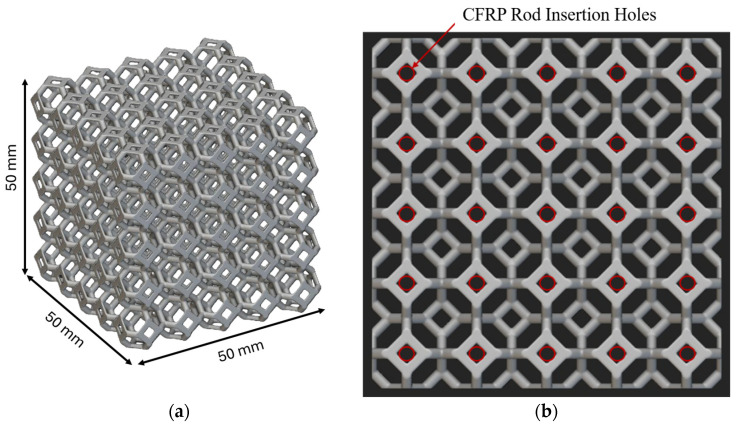
Geometrical representation of the Kelvin-cell lattice specimen: (**a**) three-dimensional view of the designed lattice structure, and (**b**) top-view schematic indicating the circular lattice holes used as CFRP rod insertion openings.

Kelvin-cell lattices were designed using nTopology software version 4.6.2, comprising cubic unit cells with a side length of 10 mm and circular struts with a diameter of 1.5 mm. Each specimen was a 50 mm cube composed of 5 × 5 × 5 repeated cells (Figure 2a). The Kelvin-cell specimens incorporated five repeated unit cells along each principal direction to mitigate boundary and size effects during compression testing. This design choice was based on previous studies indicating that lattice specimens require a sufficient number of repeated unit cells, commonly at least four in each direction, to yield a representative mechanical response [33,34].

The Kelvin-cell lattices were fabricated from Anycubic Tough Resin 2.0 (Shenzhen Anycubic Technology Co., Ltd., Shenzhen, China) using a Phrozen Sonic Mega 8K masked stereolithography printer (Phrozen Tech Co., Ltd., Hsinchu City, Taiwan). The resin was selected as the base photopolymer because of its balanced mechanical performance, including a tensile strength of 30–42 MPa and an elongation at break of 60–72% [35]. Its relatively high ductility was expected to reduce the brittle failure commonly observed in standard photopolymer resins under compression. The physical and mechanical properties of the resin are summarized in Table 1. The specimens were fabricated using a Phrozen Sonic Mega 8K MSLA printer with the process parameters listed in Table 2. Following fabrication, the specimens were cleaned in a 95% isopropyl alcohol solution for 10 min to remove residual uncured resin, before being post-processed in accordance with the manufacturer’s recommendations.

### 2.2. CFRP Rod-Reinforced Kelvin-Cell Lattice Specimen Preparation

The CFRP rod-reinforced specimens were prepared following the fabrication of the polymeric Kelvin-cell lattices. The rods were manually inserted into designated longitudinal cavities according to the experimental design. Both the insertion openings and the commercially supplied unidirectional pultruded CFRP rods shared a nominal diameter of 2 mm. Pultruded unidirectional CFRP rods with a diameter of 2 mm were purchased from Dost Kimya End. Ham. Ltd. Şti. (Istanbul, Türkiye). The rods were therefore assembled under a manually achievable close-fit condition. No adhesive was applied at the rod–lattice interface, thereby enabling the direct evaluation of the rods’ mechanical contribution without any confounding bonding effects. In all reinforced configurations, the rods were inserted from the bottom surface and maintained in direct contact with the lower compression platen. Load transfer was consequently governed by axial contact, lateral confinement from the surrounding lattice openings, and progressive interaction between the rods and the deforming polymeric matrix. The physical and mechanical properties of the CFRP rods as reported in the supplier’s technical data sheet, are presented in Table 3. Rods of varying lengths were cut and inserted into the designated openings shown in Figure 2b to achieve the required reinforcement patterns and configurations.

The design of the CFRP rod-reinforced hybrid Kelvin-cell lattice specimens was defined by three main variables: (i) the rod placement pattern group, (ii) the number of rods, and (iii) the rod-length configuration (Table 4). Three different placement pattern groups, denoted as P1, P2, and P3, were evaluated to examine the influence of rod distribution pattern within the Kelvin-cell lattice. The P1 group was characterized by a core-dominated reinforcement layout, wherein the CFRP rods were primarily concentrated within the central, load-bearing region of the specimen. The P2 group, on the other hand, followed a frame-type arrangement, in which the rods were positioned predominantly along the outer regions and corners of the lattice structure. Finally, the P3 configuration represented a distributed architecture, where the rods were arranged uniformly across the specimen’s cross-section. For each placement group, two rod numbers were used: five (N5) and thirteen (N13), corresponding to specimens reinforced with 5 and 13 CFRP rods, respectively. Furthermore, five rod-length configurations were evaluated: U30, U40, U50, 50/50, and G. In the uniform (U-type) configurations, the CFRP rod length was varied with respect to the total specimen height. Given that the overall specimen height was 50 mm, rod lengths of 30 mm, 40 mm, and 50 mm corresponded to 60%, 80%, and 100% of the specimen height, respectively. Accordingly, U30, U40, and U50 represented specimens reinforced with 30 mm, 40 mm, and 50 mm CFRP rods. For the hybrid configuration, denoted as 50/50, short (U30) and long (U50) CFRP rods were incorporated simultaneously within the same specimen. Additionally, a graded configuration, referred to as G, was introduced by integrating short (U30), medium (U40), and long (U50) rods into the same reinforcement architecture, aiming to provide a functionally graded reinforcement distribution across the specimen’s cross-section. These configurations were designed to systematically investigate the effects of rod placement pattern, the number of rods, and rod-length configuration on the deformation sequence, mechanical response, and crashworthiness of the CFRP rod-reinforced Kelvin-cell lattice structures.

The placement patterns and rod-length configurations are schematically illustrated in Table 4. The symbols S, M, and L denote rod lengths of 30, 40, and 50 mm, respectively. In addition, ○ denotes an unreinforced unit cell of the Kelvin-cell lattice. The full factorial design consisted of three placement patterns, two rod-number levels, and five rod-length configurations, yielding 30 different reinforced conditions. The specimens were coded using the format PN-C, where P, N, and C denote the placement pattern, the number of rods, and the rod-length configuration, respectively. For instance, the nomenclature P1N5-U30 identifies a P1 specimen reinforced with five 30 mm CFRP rods. In all configurations, the rods were inserted from the bottom surface and maintained in direct contact with the lower platen, with the remaining upper gap being directly dependent on the respective rod length.

### 2.3. Mechanical Tests and Evaluation

The quasi-static compression tests were conducted in accordance with the general principles of the ISO 844 standard [37]. All specimens had identical dimensions of 50 mm × 50 mm × 50 mm and were compressed between two rigid parallel plates under displacement-controlled loading. The loading direction was aligned with the vertical axis of the lattice structure and the CFRP rods. The tests were performed using an Instron 3382 universal testing machine equipped with a 100 kN load cell at a constant crosshead displacement rate of 5 mm/min under ambient conditions. Each specimen was centered on the lower platen to minimize eccentric loading, with force–displacement data being continuously recorded at a sampling frequency of 100 Hz. The compression tests continued to a nominal compressive strain of 50%, corresponding to a platen displacement of 25 mm. A minimum of three replicate tests were performed for each design group to ensure statistical repeatability.

The resulting force–displacement curves were used as the primary experimental output for the crashworthiness evaluation. Based on these curves, key performance parameters including energy absorption, specific energy absorption, peak crushing force, initial peak crushing force, mean crushing force, and crushing force efficiency parameters were calculated. The crashworthiness performance of the empty and hybrid (CFRP rod-reinforced) Kelvin-cell lattice structures was evaluated using these parameters derived from the experimental force–displacement curves. Unreinforced Kelvin-cell lattice specimens were also tested to serve as a pure polymeric group. These control specimens, denoted as KC-E, featured the same overall dimensions and lattice architecture as their reinforced counterparts. Consequently, the mechanical response and crashworthiness metrics of the CFRP rod-reinforced specimens were evaluated relative to this unreinforced baseline.

The energy absorption (EA) was calculated as the area under the force–displacement curve over the deformation distance (Equation (1)) [38], where F(s) is the crushing force and s is the deformation displacement. Other important crashworthiness parameters, namely, SEA, MCF, CFE_IPCF_, and CFE_PCF_, were obtained according to Equations (2)–(5) [38], respectively. SEA was obtained by normalizing EA by the specimen mass (m). The mean crushing force (MCF) was used as a key crashworthiness parameter to quantify the force level sustained during the energy absorption process over the deformation distance. Accordingly, MCF was determined by dividing the total absorbed energy by the corresponding deformation displacement. In this study, the crushing force efficiency (CFE) was assessed based on two different reference forces. The peak crushing force (PCF) was defined as the highest crushing force obtained during the deformation, while the initial peak crushing force (IPCF) was defined as the first peak force recorded at the early stage of deformation.

(1) $$\mathrm{EA}=\int_{0}^{s}F\left(s\right)\mathrm{ds}$$

(2) $$\mathrm{SEA}=\frac{\mathrm{EA}}{m}$$

(3) $$\mathrm{MCF}=\frac{\mathrm{EA}}{s}$$

(4) $${\mathrm{CFE}}_{\mathrm{PCF}}=\frac{\mathrm{MCF}}{\mathrm{PCF}}$$

(5) $${\mathrm{CFE}}_{\mathrm{IPCF}}=\frac{\mathrm{MCF}}{\mathrm{IPCF}}$$

### 2.4. Data-Driven Assessment

A data-driven regression approach was adopted to evaluate the relationship between the reinforcement variables and the crashworthiness responses. The main objective of the analysis was to assess the relative influence of design parameters defined in the experimental program on the key performance metrics. In this context, the reinforcement placement pattern, the number of CFRP rods, and the rod-length configurations were used as input variables, whereas SEA, CFE_IPCF_, and CFE_PCF_ were considered as target responses. The primary objective was to quantify the relative influence of these individual design factors within the experimental design space.

The regression models were developed using MATLAB R2024b, based on the experimental results obtained from the quasi-static compression tests. Several regression model families available in MATLAB Regression Learner were trained and compared, including regression trees (RT) [39], support vector machines (SVMs) [40], Gaussian process regression (GPR) [41,42], ensemble regression, and artificial neural network (ANN) [43]. Fivefold cross-validation was used to assess predictive performance and mitigate the risk of overfitting.

The models were compared using standard regression performance indicators, namely root mean square error (RMSE), mean absolute error (MAE), and the coefficient of determination (R-squared (R^2^)). For each target response, the most suitable model was selected by simultaneously evaluating both the RMSE and the corresponding R^2^ values. RMSE indicates the average prediction error magnitude by comparing predicted values with their corresponding experimental values [44]. Furthermore, Bayesian optimization was employed to systematically tune the hyperparameters of the optimizable regression models, where the configuration yielding the best cross-validation performance was retained as the optimal parameter set. The trained models were subsequently utilized as a complementary tool to interpret the experimental results and to assess the influence of the reinforcement design parameters. All model-based interpretations were limited to the investigated design space.

A three-way analysis of variance (ANOVA) was conducted to evaluate the statistical significance and relative contribution of the placement pattern, rod number, and rod-length configuration on SEA, CFE_IPCF_, and CFE_PCF_. The analysis was supported by the fully crossed factorial structure of the experimental program, which comprised three placement patterns, two rod numbers, and five rod-length configurations. With three replicates for each factor combination, this setup resulted in a total of 90 specimens. A full factorial ANOVA model incorporating all main effects and their multi-way interactions was evaluated using Type-II sums of squares. Effects were considered statistically significant at *p* < 0.05, corresponding to a 95% confidence level. The percentage contribution of each term was calculated by dividing its partial sum of squares by the total sum of squares and multiplying by 100. Consequently, the ANOVA complemented the data-driven main-effects analysis by providing a robust, inferential assessment of the design factors [45].

## 3. Results and Discussion

### 3.1. Force–Displacement Characteristics of Empty and CFRP Rod-Reinforced Kelvin-Cell Lattices

**Figure 3:**
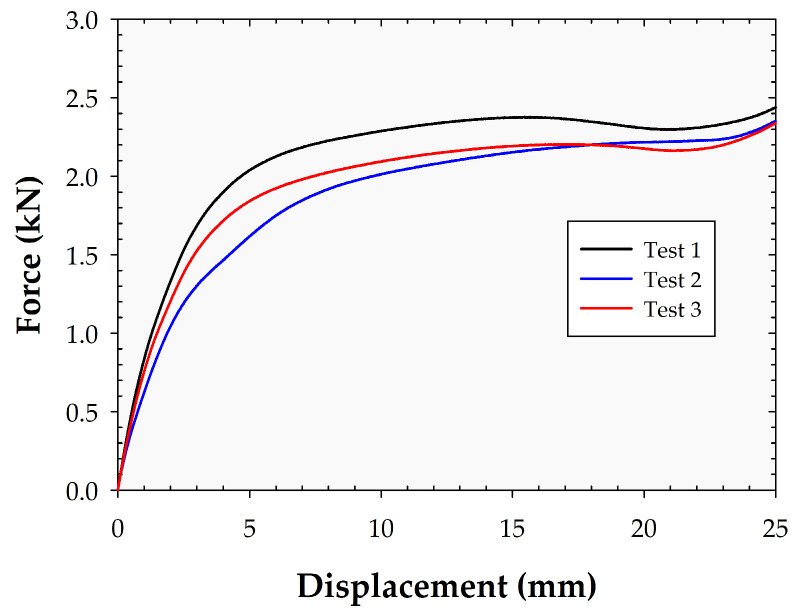
Force–displacement curves of the KC-E specimens obtained from three repeated quasi-static compression tests.

The force–displacement responses were first examined to clarify the effect of CFRP rod reinforcement on the resulting crushing behavior. Figure 3 presents the force–displacement curves obtained from three repeated compression tests of the empty Kelvin-cell lattice (KC-E), which served as the baseline. The KC-E specimens exhibited a progressive increase in force, followed by a relatively stable load-carrying plateau without an abrupt force drop. In the initial stage of compression, the force increased rapidly up to approximately 2–5 mm of displacement, which is attributable to the elastic deformation of the constituent Kelvin-cell struts. Beyond this elastic regime, the slope of the curves gradually decreased, and the force response approached a plateau-like trend. This indicates that the KC-E specimens underwent progressive crushing, with the load level remaining within the range of 2.0–2.4 kN throughout the later stages of deformation, thereby confirming a highly stable progressive folding mechanism.

The crashworthiness performance metrics of the KC-E specimens are summarized in Table 5. The KC-E specimens absorbed an average energy of 49.73 J during compression, corresponding to an SEA value of 1.95 J/g. The MCF of the empty lattice was calculated as 1989.21 N. Accordingly, the CFE_IPCF_ and CFE_PCF_ values were determined to be 87.9% and 83.7%, respectively. These relatively high CFE values indicate that the empty Kelvin-cell lattice exhibited a stable crushing response, with the mean crushing force remaining close to both the initial peak and maximum peak force levels.

**Figure 4:**
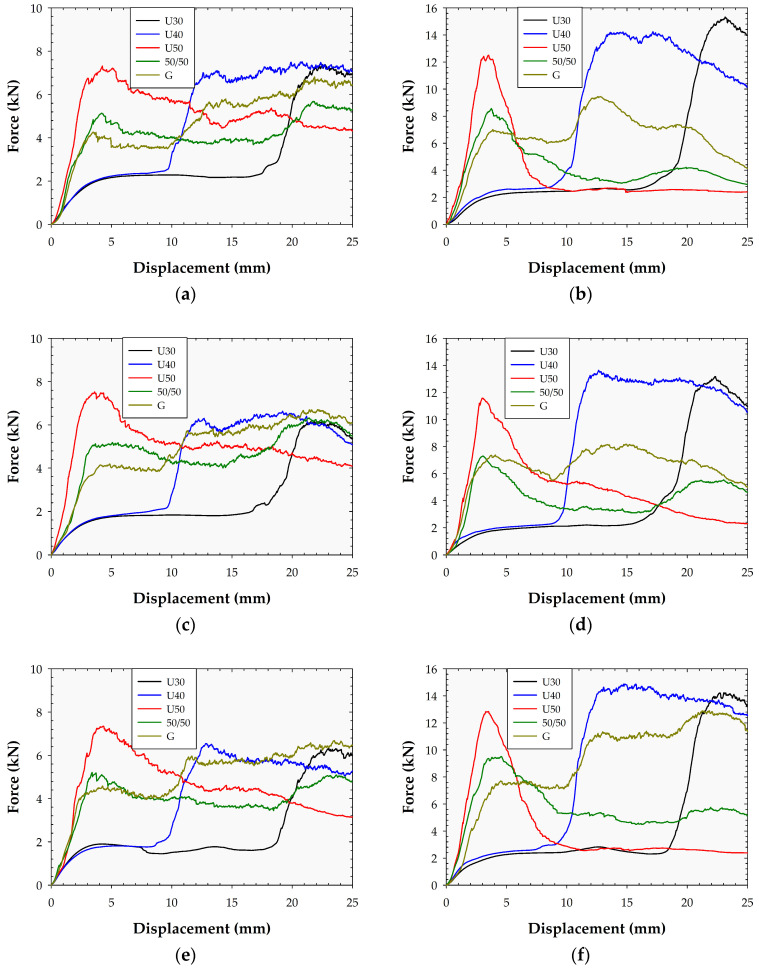
Comparison of force–displacement curves for different configurations and rod number: (**a**) P1N5, (**b**) P1N13, (**c**) P2N5, (**d**) P2N13, (**e**) P3N5, and (**f**) P3N13.

Figure 4 shows the average force–displacement curves of the hybrid Kelvin-cell lattice configurations based on three repeated compression tests. The CFRP rod-reinforced specimens exhibited a markedly higher load-bearing capacity than the empty Kelvin-cell lattice. Figure 4 also indicates that the hybrid lattice specimens presented distinct force–displacement characteristics depending on the placement pattern, number of rods, and rod-length configuration.

Among the uniform rod-length configurations, the U50 specimens exhibited the highest initial stiffness and the most pronounced initial peak force. This behavior stemmed from the immediate engagement of the full-height rods, which carried a substantial portion of the axial load before significant lattice deformation occurred. Following the initial peak, particularly in the N13 groups, the force decreased sharply as the rods underwent axial instability and lateral buckling once the deforming polymeric lattice could no longer maintain their alignment. Similar compressive failure mechanisms, including Euler-type instability, buckling, fiber splitting, matrix cracking, and fiber/matrix debonding have been reported for pultruded carbon-fiber/epoxy rods and composite lattice elements [21,26,46]. In contrast, the U30 and U40 configurations exhibited delayed reinforcement engagement. The U30 specimens demonstrated the latest activation, characterized by a marked force increase only after approximately 20 mm of displacement. Meanwhile, the U40 configuration yielded a more sustained response, wherein the force increased at intermediate displacements and remained high over a broad crushing range. The graded configuration generated a stepwise force increment due to the sequential engagement of the rods of varying lengths. This progressive engagement was particularly evident in the P3 group, thereby promoting a stable increase in crushing resistance without an excessive initial peak. These observations are consistent with previous findings indicating that reinforcement architecture, fiber arrangement, and hybridization strategies strongly influence the deformation and energy-absorption behavior of carbon-fiber-reinforced lattice structures [21,26,32].

Increasing the rod number from 5 to 13 generally elevated the overall load response; however, the magnitude of this effect was strongly dependent on both rod-length configuration and the placement pattern. The U40 and G configurations benefited most from the higher rod number, maintaining elevated force levels over a wider displacement range. These findings show that crashworthiness was governed not only by reinforcement quantity but also by the engagement length and spatial distribution of the rods. The P3 pattern provided a comparatively balanced response when combined with the graded configuration. Overall, the force–displacement results confirm that CFRP rod reinforcement is an effective strategy for enhancing the crashworthiness of Kelvin-cell lattice structures. Crucially, the selection of an optimal reinforcement architecture must not rely solely on the maximum crushing force. Instead, a holistic evaluation framework incorporating the initial peak force, post-peak stability, progressive deformation behavior, and total energy-absorption capacity must be implemented.

### 3.2. Effects of Placement Pattern, Rod Number, and Rod-Length Configuration on Crashworthiness Metrics

**Figure 5:**
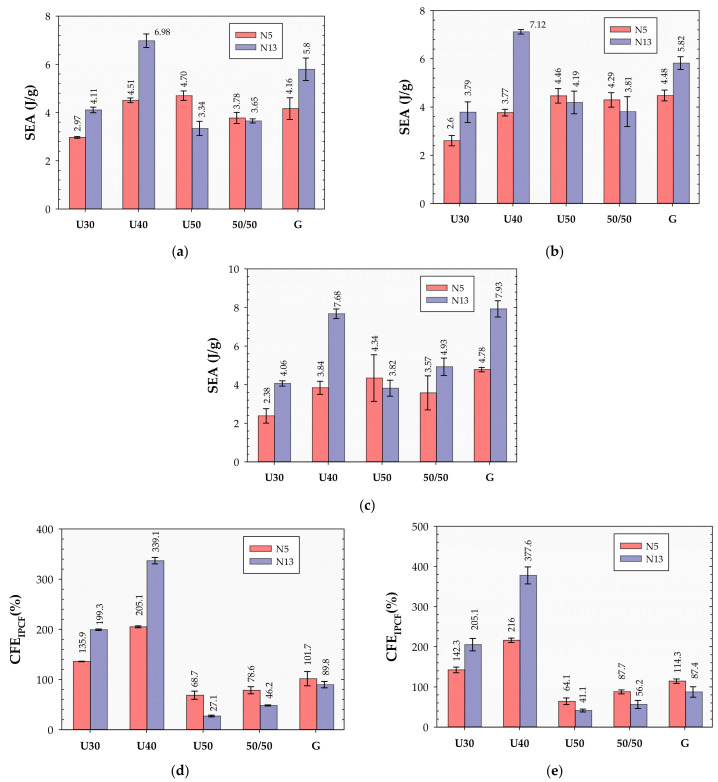

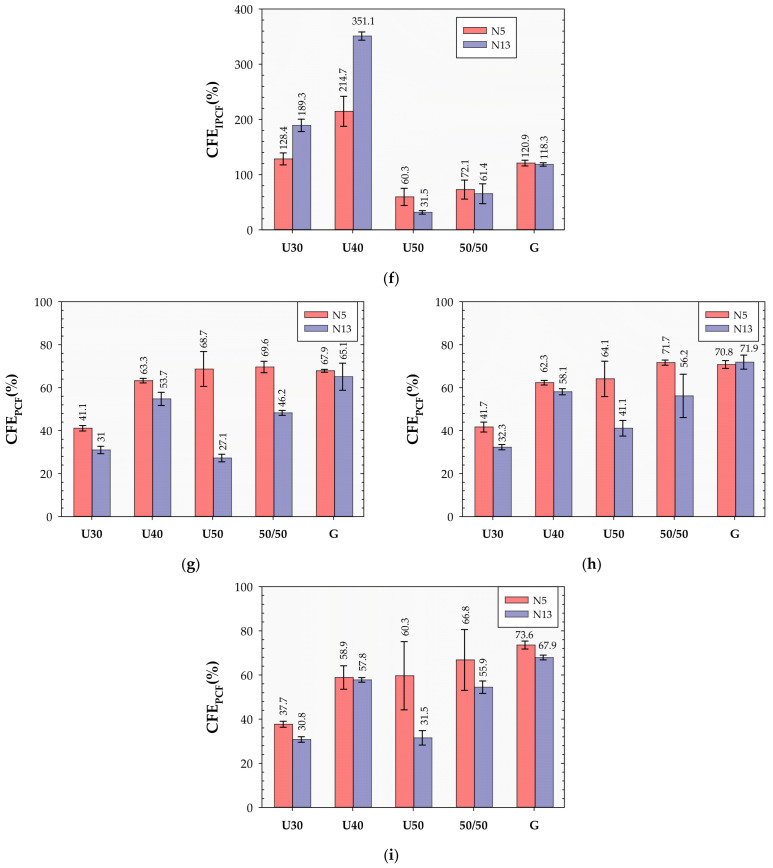
Comparison of crashworthiness metrics for different specimen groups, rod numbers and configurations. SEA: (**a**) P1, (**b**) P2, and (**c**) P3; CFE_IPCF_: (**d**) P1, (**e**) P2, and (**f**) P3; CFE_PCF_: (**g**) P1, (**h**) P2, and (**i**) P3.

The quantitative crashworthiness metrics calculated from the force–displacement curves are summarized in Table 5. Additionally, obtained crashworthiness parameters such as SEA, CFE_IPCF_, and CFE_PCF_ for different hybrid specimens are presented in Figure 5 as comparative error-bar plots with standard deviations. As indicated in Table 5, all hybrid Kelvin-cell lattice specimens exhibited superior crashworthiness compared to the empty specimens in terms of energy absorption, specific energy absorption, and mean crushing force. This result confirms the beneficial role of the vertically inserted CFRP rods in improving the axial crushing response of the lattice architecture. Furthermore, this substantial improvement indicates that the inserted CFRP rods enhanced both the load-bearing capacity and the energy absorption capability of the Kelvin-cell structure (Table 5). Specifically, they P3N13-G and P3N13-U40 hybrid specimens exhibited the highest energy absorption capacity, reaching 226.7 J and 221 J, respectively. Also, their corresponding SEA values were 7.93 J/g and 7.68 J/g (Figure 5). The superior performance of these configurations can be attributed to the combined effect of number of rods, rod placement pattern, and effective rod-length configuration during compression. Meanwhile, the G and U40 configurations promoted a more efficient energy absorption performance during crushing across all placement groups. Among the uniform length configurations, the N13-U40 specimen groups demonstrated a particularly balanced crashworthiness profile owing to their delayed but sustained load-bearing behavior, yielding elevated MCF, CFE, and SEA values. These mechanical responses are advantageous because they enable higher energy absorption without an excessively high initial peak force. For the U50 configuration, specimens produced higher initial peak force levels; however, after the initial peak force, force levels were dramatically reduced and specimens exhibited lower SEA values, especially in the N13 rod number series. Therefore, these results demonstrate that an effective reinforcement design must provide stable post-peak behavior and progressive energy absorption rather than maximizing the initial force.

The standard deviations in Table 5 varied considerably among the reinforcement configurations, indicating that repeatability was dependent on the governing deformation mechanism. The highest dispersion occurred in configurations involving early rod engagement and pronounced buckling-related instability, particularly P3N5-U50 and P3N5-50/50. The buckling and post-buckling behavior of the CFRP rods may be sensitive to small specimen-to-specimen variations in rod straightness, alignment, insertion condition, rod-lattice contact, local imperfections in the printed lattice, and defects in the pultruded rods. Such variations can alter the onset, direction, and progression of instability. Because EA and SEA are calculated by integrating the entire force–displacement response, differences in the post-peak collapse can produce substantial variations in the calculated energy-absorption metrics. In contrast, configurations characterized by delayed or sequential rod engagement and progressive lattice compaction, such as P3N13-G and P3N13-U40, exhibited markedly lower dispersion. Thus, U40 and G provided not only high SEA but also more stable and repeatable crushing responses, whereas U50 and selected 50/50 configurations were more sensitive to rod instability and post-peak variability.

Figure 5 demonstrates that crashworthiness was governed by rod-length configuration and placement pattern as well as rod number. In general, increasing the rod number from 5 to 13 improved SEA mainly for U30, U40, and G configurations, whereas U50 and 50/50 configurations did not always benefit from this increase. This indicates that the effectiveness of CFRP reinforcement cannot be evaluated only based on the number of rods. For the P1 and P2 placement pattern groups, the highest SEA values were obtained from the N13-U40 configurations. In the P3 placement pattern group, the graded configuration with 13 CFRP rods (N13-G) offered the highest SEA value. In terms of SEA, U40 and G configurations provide a desirable balance between specific energy absorption capacity and crushing force efficiency in all placement pattern groups. For the CFE_IPCF_ metric, the results showed that N13-U40 specimen groups exhibited high values in all placement pattern groups. The high CFE_IPCF_ metrics are related to the progressive activation of the CFRP rods during the compression. In contrast, the CFE_PCF_ metric provided a more conservative evaluation of the crushing stability. G and 50/50 hybrid specimens generally exhibited more balanced responses than U40 and U50. Especially in the P3 group, the G hybrid specimens showed high CFE_PCF_ values while also maintaining competitive SEA values. These findings indicate that the graded CFRP reinforcement strategy reduced the adverse effects of excessive peak force and offered a more stable crushing response. Considering the crashworthiness parameters together, it can be concluded that the U40 configuration is highly effective in improving SEA and CFE_IPCF_ due to delayed reinforcement effects, whereas the G configuration provides a more balanced response in terms of SEA and CFE_PCF_. Among all investigated configurations, P3N13-G appears to provide the most advantageous overall crashworthiness performance, while P3N13-U40 also represents a strong candidate for applications prioritizing high specific energy absorption and mean crushing force.

### 3.3. Deformation Behavior of Empty and Hybrid Kelvin-Cell Lattice Specimens

**Figure 6:**
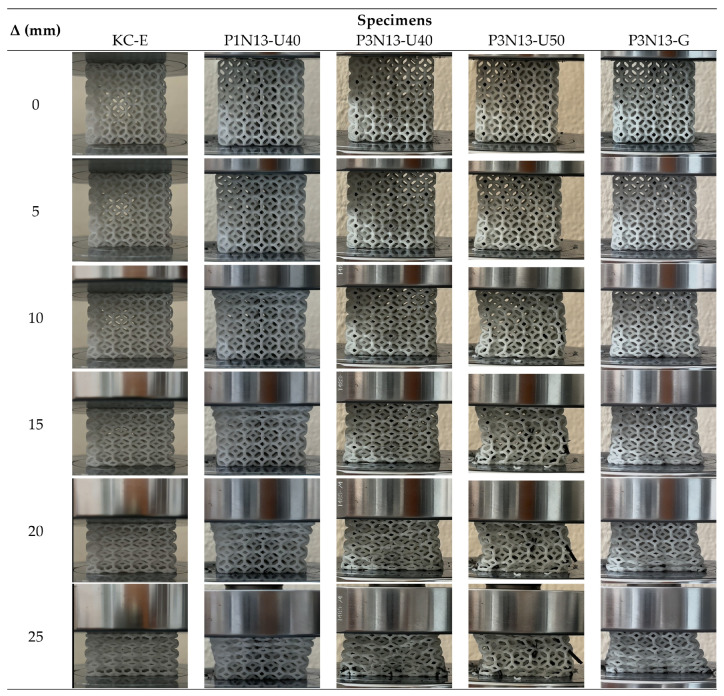
Progressive deformation sequences of selected Kelvin-cell lattice specimens under quasi-static compression at different displacement levels: KC-E, P1N13-U40, P3N13-U40, P3N13-U50, and P3N13-G.

The progressive deformation sequences of selected specimens are presented in Figure 6 to clarify the deformation mechanisms responsible for the observed crashworthiness responses. As seen from Figure 6, the empty specimen showed a relatively uniform and progressive cell-collapse mode without catastrophic failure. This behavior is consistent with its force–displacement characteristics as previously shown in Figure 3.

For the U40 configurations with P1 and P3 placement patterns, it can be seen that the deformation mechanism altered depending on the rod placement pattern. In the P1 placement pattern reinforced with U40 CFRP rods, deformation initiated in the upper cells and progressed through cell opening and crushing. In contrast, in the P3 placement pattern, deformation proceeded in a more stable and orderly manner, accompanied by the opening and crushing of the lower cells. This difference in the rod placement pattern resulted in distinct force–displacement characteristics, and the U40 configuration exhibited one of the highest SEA values among the investigated configurations. Therefore, these results indicate that both the arrangement and the length of the CFRP rods had a considerable influence on the deformation mechanism and the characteristic mechanical response. For the P3N13-U50 specimen, the deformation sequence indicates that the load-carrying of full-height CFRP rods began at the beginning of compression. Although this early activation increased the initial force level, the surrounding polymer lattice progressively deformed and could no longer provide sufficient lateral support to maintain the vertical alignment of the rods. As a result, the CFRP rods experienced local bending and buckling, followed by a rapid reduction in their axial load-carrying capacity. This instability mechanism is consistent with the compressive failure characteristics of carbon-fiber-reinforced structures, where buckling, fiber splitting, matrix damage, and loss of alignment can lead to sudden post-peak load reduction [21,26,46]. Therefore, the sudden force drop observed after the initial peak in the U50 configuration can be attributed to premature rod engagement, high peak force formation, and subsequent rod instability. Furthermore, for example, the images provided for the U50 configuration with deformation distances of 10 and 15 mm show that the deformation mechanism occurs with lateral bending and buckling of the CFRP rods.

In contrast, the P3N13-G specimen exhibited a more balanced deformation sequence without abrupt failure. The graded arrangement distributed reinforcement engagement over a wider displacement range, resulting in high EA, SEA, and MCF while maintaining a higher CFE_PCF_ compared with U50 (Table 5). The deformation images therefore confirm that the favorable response of P3N13-G resulted from the combined effects of rod number, graded engagement, and progressive lattice collapse.

### 3.4. Data-Driven Assessment of Crashworthiness Performance

**Figure 7:**
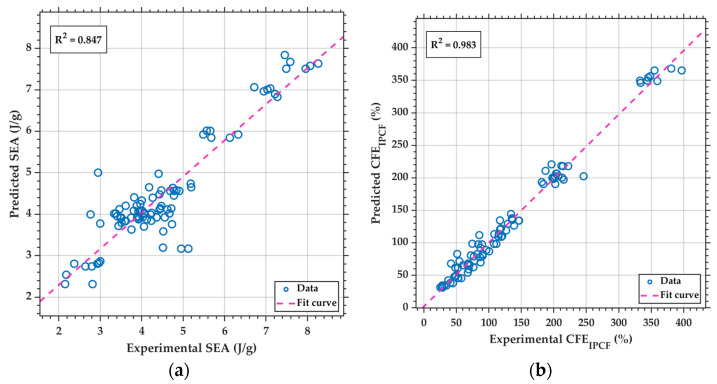

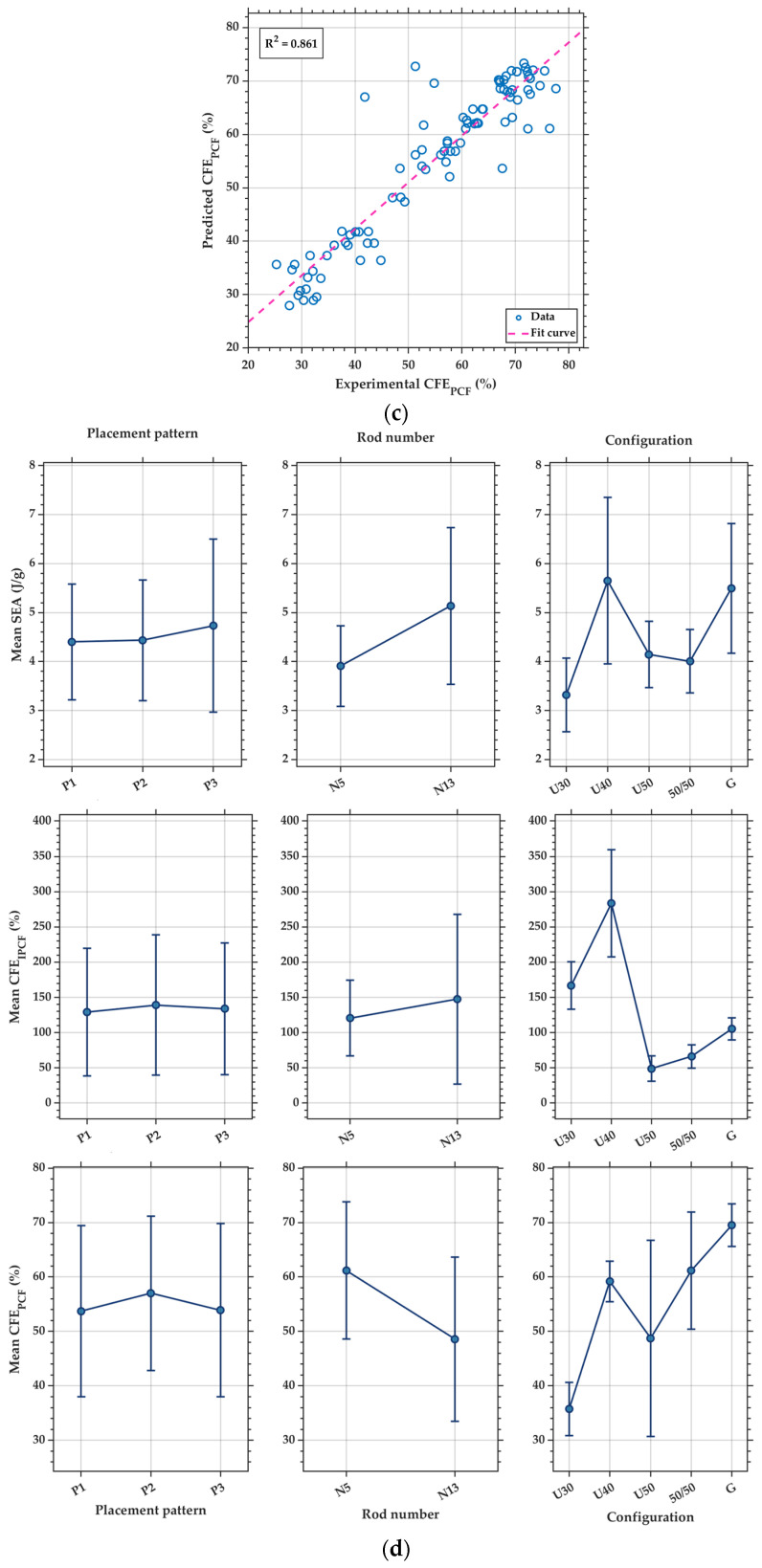
(**a**–**c**) Predicted versus experimental plots obtained using fivefold cross-validation and (**d**) main-effects plots for SEA, CFE_IPCF_, and CFE_PCF_.

A data-driven assessment was conducted to further examine the relationship between reinforcement variables and crashworthiness responses. GPR was selected as the best-performing model based on fivefold cross-validation results in Table 6 and was used for predicted-experimental comparison, main-effects analysis, and three-factor response mapping. The aim of this analysis was not only to predict experimental responses but also to identify the relative influence of the design variables on the crashworthiness metrics. The cross-validated predictive performance metrics of the GPR models are given in Figure 7a–c. The models showed good agreement with the experimental results for SEA, excellent agreement for CFE_IPCF_, and satisfactory agreement for CFE_PCF_.

The main effects plots further clarified the average influence of each design factor on the crashworthiness responses (Figure 7d). For SEA, rod-length configuration and number of rods were the most influential factors, while placement pattern had a weaker but still noticeable effect. U40 yielded the highest mean SEA, followed by the G configuration, and increasing the rod number from 5 to 13 improved SEA by enhancing the load-carrying and energy absorption capacity. Among the placement patterns, P3 provided a slightly higher mean SEA than the P1 and P2 groups, consistent with the superior SEA values of P3N13-G and P3N13-U40. For CFE_IPCF_, reinforcement configuration was the dominant factor, with U40 showing the highest mean value, followed by U30, whereas U50 produced the lowest response. This indicates that CFE_IPCF_ was mainly controlled by the length of the CFRP rods. In the U30 and U40 configurations, the rods became more effective after partial lattice deformation, allowing the mean crushing force to exceed the initial peak force, while the full-height U50 rods were engaged from the beginning and generated a high IPCF. In contrast, CFE_PCF_ showed a different trend. Increasing the number of rods reduced mean CFE_PCF_ because the higher number of rods increased the overall peak crushing force more strongly than the mean crushing force. Among the configurations, G provided the highest mean CFE_PCF_, followed by 50/50 and U40, suggesting that the graded reinforcement layout offered a more favorable balance between mean crushing force and peak force.

The ANOVA results for SEA, CFE_IPCF_, and CFE_PCF_ are presented in Table 7 and quantitatively support the factor ranking obtained from the data-driven main-effects analysis. For all three responses, the rod-length configuration is the dominant and highly significant factor, explaining 41.6% of the variance for SEA (F = 109.6, *p* < 0.001), 83.2% for CFE_IPCF_ (F = 1496.6, *p* < 0.001), and 59.2% for CFE_PCF_ (F = 110.7, *p* < 0.001). The rod number is a significant secondary factor, contributing 19.1% to SEA and 17.4% to CFE_PCF_ (*p* < 0.001 in both cases). Importantly, the configuration x rod-number interaction is also significant for all responses (24% for SEA, 13% for CFE_IPCF_, and 11.6% for CFE_PCF_; *p* < 0.001), which provides a formal statistical basis for the observation that the benefit of increasing the rod number depends on the rod-length configuration. Placement pattern showed the lowest percentage contribution for all three responses; nevertheless, its main effect remained statistically significant for SEA, CFE_IPCF_, and CFE_PCF_ (*p* < 0.05). The significant three-way interaction for SEA indicates that the effect of placement pattern varied jointly with rod number and rod-length configuration. Therefore, the main-effect contributions for SEA represent average tendencies and should be interpreted together with the configuration-specific experimental trends. The full factorial model accounts for almost all of the observed variance (R^2^ = 0.94 for SEA, 0.99 for CFE_IPCF_, and 0.92 for CFE_PCF_), confirming that the three design variables and their interactions capture the crashworthiness behavior well. These results agree with the data-driven model and reinforce the conclusion that the rod-length configuration is the principal design lever for the crashworthiness of CFRP-rod-reinforced Kelvin-cell lattices, with the rod number acting as a significant secondary factor through its interaction with the configuration.

**Figure 8:**
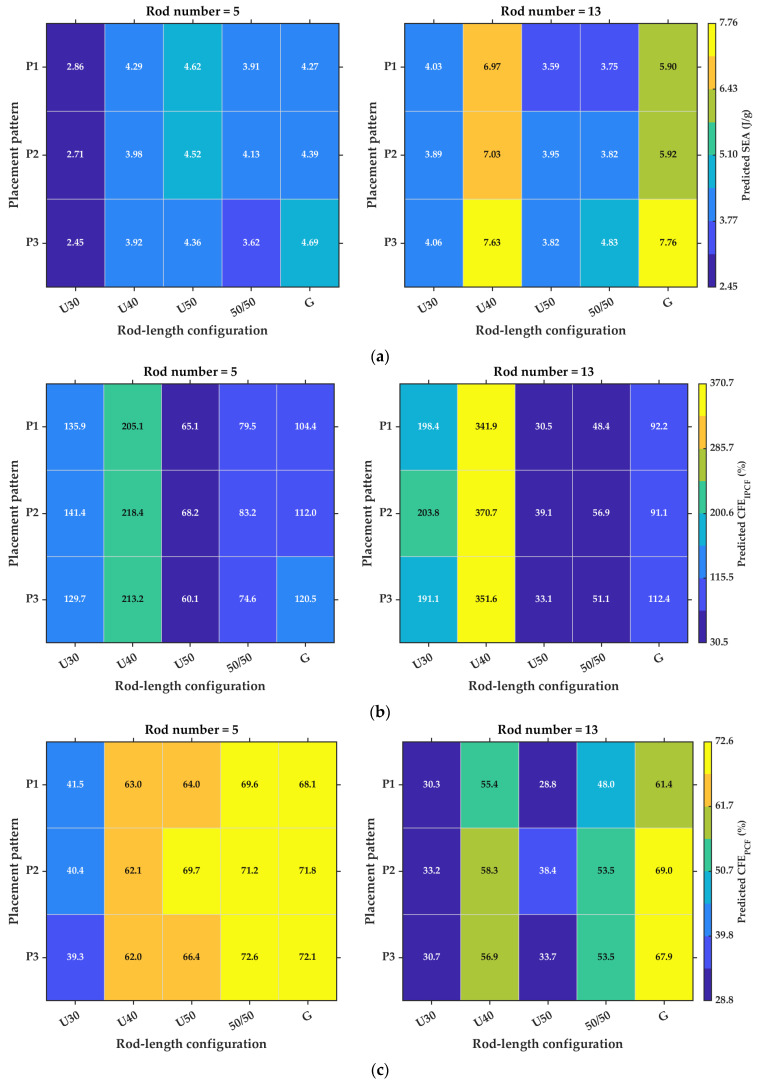
Response maps of crashworthiness parameters: (**a**) SEA, (**b**) CFE_IPCF_, and (**c**) CFE_PCF_.

Figure 8 visualizes the combined effects of placement pattern, rod number, and rod-length configuration on SEA, CFE_IPCF_, and CFE_PCF_. For SEA, the highest predicted response region was associated with N13 specimens combined with U40 and G configurations. In particular, P3N13-G and P3N13-U40 appeared as the most favorable combinations, which agrees well with the experimental results, where these specimens exhibited the highest SEA and MCF values. For CFE_IPCF_, the U40 configuration consistently formed the highest response region for all placement patterns and rod numbers, especially in the N13 group. For CFE_PCF_, however, the highest response regions were concentrated around the G and 50/50 configurations. This indicates that these configurations provide a better balance between mean crushing force and peak crushing force.

Overall, crashworthiness could not be optimized using a single design variable. Instead, each response parameter was governed by a different combination of reinforcement characteristics. The U40 length configuration was particularly advantageous for improving SEA and CFE_IPCF_, mainly because the CFRP rods became highly effective after partial lattice deformation, producing an effective load-carrying contribution. In contrast, the G configuration offered a more balanced mechanical response by maintaining high SEA while also improving CFE_PCF_. Although increasing the rod number generally enhances energy absorption and mean load-bearing capacity, it could also increase the peak crushing force, thereby reducing efficiency when normalized by PCF. Therefore, the determination of the optimal reinforcement design should depend on the targeted crashworthiness requirement. Within the investigated design space, P3N13-G was more suitable for maximizing SEA and MCF, whereas P3N13-U40 provided the most favorable overall balance among the evaluated metrics.

From an engineering perspective, the proposed CFRP rod-reinforced Kelvin-cell lattice concept offers a tunable lightweight energy-absorbing structure. By adjusting placement pattern, rod number, and rod-length configuration, the balance among SEA, MCF, peak-force control, and deformation stability can be tailored to the target application. Therefore, configurations such as P3N13-U40 may be preferred when high SEA and MCF are required, whereas P3N13-G may be more suitable for applications requiring a balanced crashworthiness response. These characteristics indicate that the proposed hybrid lattice structures may be promising for lightweight protective cores, crash absorbers, cushioning systems, transport-related impact-protection components, and sandwich-type structural cores. Beyond the specific designs, two features of the proposed approach are attractive from an engineering standpoint: the reinforcement is introduced by a simple, adhesive-free press-fit that requires no change to the printing process and allows the rods to be replaced or recovered, and the crashworthiness response can be tuned over a wide range by selecting the rod number, placement pattern, and rod-length configuration. This tunability allows a single lattice platform to be adapted to different energy-absorption and peak-force targets without redesigning the unit cell.

## 4. Conclusions

This study experimentally investigated the crashworthiness enhancement of polymer Kelvin-cell lattice structures through discrete CFRP rod reinforcement under quasi-static compression. Regression-based modelling and three-way factorial ANOVA were additionally used to evaluate the individual and interactive effects of placement pattern, rod number, and rod-length configuration. The following conclusions were drawn from the force–displacement responses, deformation sequences, crashworthiness metrics, predictive models, and statistical analyses:

- Compared with the empty Kelvin-cell lattice, the CFRP rod-reinforcement strategies increased EA, SEA, and MCF by more than 300%. These improvements demonstrate that discrete CFRP rods can substantially enhance the axial load-bearing and energy-absorption capacities of polymeric Kelvin-cell lattices without requiring modification of the original unit-cell topology.
- Rod-length configuration was the dominant design factor. Full-height U50 rods engaged immediately, producing high initial and peak forces but also early rod instability and pronounced post-peak load reduction. In contrast, U40 rods provided delayed and sustained reinforcement engagement, resulting in high SEA, MCF, and CFE_IPCF_, while the graded configuration distributed rod activation over a wider displacement range and provided a favorable balance between energy absorption and peak-force control.
- The effect of rod number depended strongly on rod-length configuration. Increasing the rod number was most beneficial for the U30, U40, and graded configurations, whereas it did not consistently improve the U50 and 50/50 responses. Placement pattern had a smaller average effect, although the distributed P3 architecture generally promoted more uniform load transfer and favorable performance when combined with N13 and the U40 or graded configurations. P3N13-G achieved the highest EA, SEA, and MCF, while U40 configurations were particularly advantageous when delayed activation and high CFE_IPCF_ were prioritized.
- The ANOVA results supported the factor ranking and response trends obtained from the data-driven models. Rod-length configuration and rod number were the principal determinants of the response, while the significant interaction effects demonstrated that the reinforcement variables should be selected jointly rather than independently.

From an engineering perspective, the CFRP rod-reinforced Kelvin-cell concept provides a tunable lightweight energy-absorbing structure for applications requiring high specific energy absorption, controlled peak force, and stable progressive deformation. These characteristics make the proposed hybrid lattice architecture promising for lightweight protective cores, crash absorbers, cushioning components, transport-related impact-protection systems, and sandwich-type structural cores.

The present findings are limited to the investigated material system, rod diameter, reinforcement levels, placement patterns, and quasi-static loading conditions. Also, the local stress distribution and internal load-transfer mechanisms were not examined numerically. Future studies should develop experimentally validated finite element models to investigate stress concentrations, rod–lattice interaction, buckling initiation, and progressive damage. Dynamic impact, fatigue, environmental durability, and life-cycle performance should also be examined.

From a sustainability perspective, the proposed CFRP reinforcement strategy may improve mechanical efficiency per unit mass by reinforcing only selected regions. However, the multi-material nature of photopolymer/CFRP hybrid lattices requires further investigation in terms of life-cycle performance, material separation, and end-of-life recycling.

## Figures and Tables

**Table 1:** Physical and mechanical properties of photopolymer resin [35].

Properties	Value
Viscosity (25 °C) (mPa-s)	450–500
Density (g/cm^3^)	1.70–1.75
Hardness (Shore)	Shore D 82–84
Tensile Strength (MPa)	30–42
Elongation to Break (%)	60–72
Flexural Strength (MPa)	42–52
Flexural Modulus (MPa)	1500–2000
Izod Impact Strength (J/m)	143

**Table 2:** MSLA process parameters used to fabricate the Kelvin-cell lattices.

Properties	Value
Layer Height (µm)	50
Normal Exposure Time (s)	2.5–3.5
Bottom Layer Count	5–6
Bottom Exposure Time (s)	35–45
Lifting Distance (mm)	6–8
Light-off Delay (s)	0.5

**Table 3:** Physical and mechanical properties of CFRP rods [36].

Properties	Value
Resin	Vinyl Epoxy
Fiber Content (wt.%)	>75
Tensile Strength (MPa) (in direction of fiber)	2080
Compressive Strength (MPa) (in direction of fiber)	1450
Elastic Modulus (GPa)	145
Density (g/cm^3^)	1.51

**Table 4:** Schematic illustration of the hybrid Kelvin-cell lattices with placement pattern groups, rod numbers, and length configurations.

Pattern	Number of Rods (N)	Configuration				
		U30	U40	U50	50/50	G
P1	5	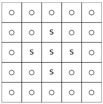	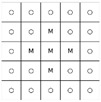	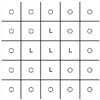	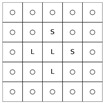	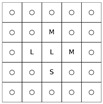
	13	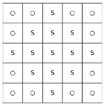	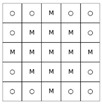	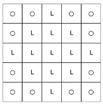	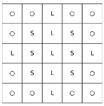	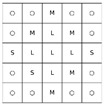
P2	5	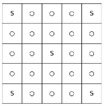	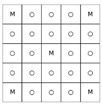	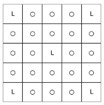	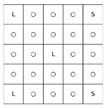	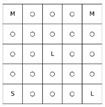
	13	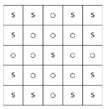	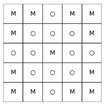	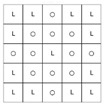	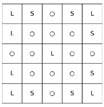	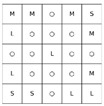
P3	5	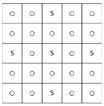	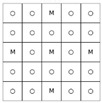	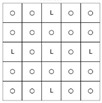	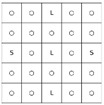	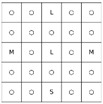
	13	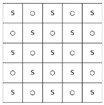	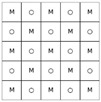	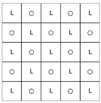	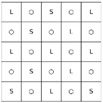	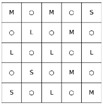

**Table 5:** Crashworthiness metrics obtained from the quasi-static compression tests.

Specimen	Crashworthiness Metrics						
	EA (J)	SEA (J/g)	IPCF (N)	PCF (N)	MCF (N)	CFE_IPCF_ (%)	CFE_PCF_ (%)
KC-E	49.7 ± 2.92	1.95 ± 0.12	2263.3 ± 92.9	2376.5 ± 53.6	1989.2 ± 117	87.9 ± 2.32	83.7 ± 3.3
P1N5-U30	77.7 ± 0.78	2.97 ± 0.04	2286.7 ± 24.9	7565 ± 117.6	3108.4 ± 31	135.9 ± 0.4	41.1 ± 1
P1N5-U40	120.1 ± 2.13	4.51 ± 0.09	2341.7 ± 45.5	7590 ± 135.7	4802.3 ± 85.2	205.1 ± 1.6	63.3 ± 0.86
P1N5-U50	127.3 ± 4.34	4.70 ± 0.19	7461.6 ± 486.1	7461.6 ± 486.1	5093.4 ± 173.8	68.7 ± 6.63	68.7 ± 6.63
P1N5-50/50	102.6 ± 5.15	3.78 ± 0.23	5239.4 ± 264.7	5909.8 ± 455.2	4105.7 ± 206	78.6 ± 5.8	69.6 ± 2.16
P1N5-G	117.5 ± 10.3	4.16 ± 0.45	4666.7 ± 532	6923.6 ± 600.6	4698.3 ± 413.2	101.7 ± 11.6	67.9 ± 0.55
P1N13-U30	117.6 ± 2.72	4.11 ± 0.12	2360 ± 49.67	15,188.6 ± 456.3	4703.5 ± 108.8	199.3 ± 1.14	31 ± 1.41
P1N13-U40	204 ± 6.7	6.98 ± 0.28	2406.7 ± 66	15,200.9 ± 540.7	8161.2 ± 267.8	339.1 ± 4.7	53.7 ± 1.9
P1N13-U50	96.1 ± 6.16	3.34 ± 0.3	14,288.6 ± 1115	14,288.6 ± 1115	3844.4 ± 246.6	27.1 ± 2.85	27.1 ± 2.85
P1N13-50/50	105.8 ± 18.15	3.65 ± 0.09	9249.2 ± 839	9249.2 ± 839	4231.3 ± 82.5	46.2 ± 4.7	46.2 ± 4.7
P1N13-G	160.2 ± 10.48	5.8 ± 0.45	7133.3 ± 179.32	9847.6 ± 131.9	6408 ± 419.4	89.8 ± 5.14	65.1 ± 5.14
P2N5-U30	65.9 ± 4.43	2.60 ± 0.21	1850 ± 58.88	6316.7 ± 142.6	2634.6 ± 177.3	142.3 ± 5.77	41.7 ± 1.9
P2N5-U40	104.8 ± 3.13	3.77 ± 0.13	1943.3 ± 97.4	6726.3 ± 180.8	4193.6 ± 125.2	216 ± 4.5	62.3 ± 0.88
P2N5-U50	125.4 ± 14.2	4.46 ± 0.3	7908 ± 872.5	7908 ± 872.5	5017.6 ± 275.8	64.1 ± 6.7	64.1 ± 6.7
P2N5-50/50	115.4 ± 6.9	4.29 ± 0.3	5263.3 ± 96.7	6444.9 ± 435.3	4615.2 ± 263.1	87.7 ± 3.9	71.7 ± 0.977
P2N5-G	122.3 ± 5.0	4.48 ± 0.23	4288.3 ± 267.6	6911.6 ± 141.9	4893.3 ± 200.8	114.3 ± 4.47	70.8 ± 1.48
P2N13-U30	107.6 ± 9.95	3.79 ± 0.43	2096.7 ± 119	13,320.7 ± 998.5	4304.3 ± 398	205.1 ± 12.6	32.3 ± 1
P2N13-U40	202.85 ± 2.13	7.12 ± 0.09	2153.3 ± 106.2	13,975.1 ± 400.6	8113.9 ± 86	377.6 ± 17.2	58.1 ± 1.16
P2N13-U50	122.76 ± 9.5	4.19 ± 0.47	11,964.9 ± 838.1	11,964.9 ± 838.1	4910.3 ± 379.8	41.1 ± 2.98	41.1 ± 2.98
P2N13-50/50	105.14 ± 14	3.81 ± 0.62	7503.9 ± 195	7503.9 ± 195	4205.4 ± 558.5	56.2 ± 8.23	56.2 ± 8.23
P2N13-G	161 ± 6	5.82 ± 0.27	7479.6 ± 902.1	8970.5 ± 413.7	6441.9 ± 240	87.4 ± 10.6	71.9 ± 2.67
P3N5-U30	62.2 ± 7.99	2.38 ± 0.37	1963.3 ± 388.7	6596 ± 720.9	2489.3 ± 319.4	128.4 ± 8.9	37.7 ± 1.13
P3N5-U40	98.9 ± 7.15	3.84 ± 0.34	1853.3 ± 147	6729.1 ± 244.2	3957.8 ± 286.2	214.7 ± 22.2	58.9 ± 4.33
P3N5-U50	113.8 ± 24.6	4.34 ± 1.21	7518.6 ± 172.6	7518.6 ± 172.6	4553.1 ± 984.1	60.3 ± 12.2	60.3 ± 12.2
P3N5-50/50	98.1 ± 19.77	3.57 ± 0.88	5491.8 ± 450.3	5907.2 ± 839.2	3925.6 ± 790.8	72.1 ± 15.2	66.8 ± 11.3
P3N5-G	125.1 ± 2.4	4.78 ± 0.11	4146.7 ± 199.1	6805.3 ± 93.3	5005.7 ± 97.4	120.9 ± 4.2	73.6 ± 1.5
P3N13-U30	112.1 ± 3.1	4.06 ± 0.14	2376.7 ± 157.6	14,569.9 ± 217.9	4484.7 ± 122.7	189.3 ± 9.3	30.8 ± 1.03
P3N13-U40	221 ± 5.8	7.68 ± 0.25	2520 ± 94.2	15,303.8 ± 402.1	8841.2 ± 232.1	351.1 ± 6	57.8 ± 0.85
P3N13-U50	104.6 ± 9.41	3.82 ± 0.41	13,446.5 ± 1470.7	13,446.5 ± 1470.7	4181.9 ± 376.4	31.5 ± 2.7	31.5 ± 2.67
P3N13-50/50	141.7 ± 10.6	4.93 ± 0.45	9413.8 ± 1655.3	10,175.3 ± 852.2	5667 ± 423.5	61.4 ± 6.8	55.9 ± 4.68
P3N13-G	226.7 ± 9.8	7.93 ± 0.42	7670 ± 449.3	13,350.6 ± 558.8	9066.1 ± 393.3	118.3 ± 2.5	67.9 ± 0.88

**Table 6:** Comparison of model performance metrics of regression models.

Model Type	RMSE			R^2^			MAE		
	SEA	CFE_IPCF_	CFE_PCF_	SEA	CFE_IPCF_	CFE_PCF_	SEA	CFE_IPCF_	CFE_PCF_
Tree	0.564	0.124	0.056	0.841	0.983	0.861	0.447	0.090	0.039
SVM	0.511	0.125	0.056	0.819	0.982	0.828	0.361	0.094	0.032
GPR	0.498	0.122	0.053	0.847	0.983	0.861	0.366	0.090	0.038
Ensemble	0.504	0.124	0.060	0.833	0.982	0.843	0.383	0.092	0.037
Neural Network	0.503	0.133	0.062	0.824	0.98	0.835	0.376	0.093	0.043

**Table 7:** ANOVA results for the crashworthiness responses.

Crashworthiness Metrics	Source	SS	df	F	*p*	% Contribution
SEA (J/g)	Pattern (P)	2.02	2	6.05	0.0041	1.15
	Rod Number (N)	33.75	1	201.90	<0.001	19.13
	Configuration (C)	73.31	4	109.63	<0.001	41.55
	P × N	5.38	2	16.09	<0.001	3.05
	P × C	6.73	8	5.03	<0.001	3.81
	N × C	42.25	4	63.19	<0.001	23.95
	P × N × C	2.96	8	2.22	0.039	1.68
	Residual (error)	10.03	60			5.68
CFE_IPCF_ (%)	Pattern (P)	1510.93	2	6.96	0.0019	0.19
	Rod Number (N)	16,276.92	1	149.85	<0.001	2.08
	Configuration (C)	650,269.91	4	1496.63	<0.001	83.20
	P × N	241.89	2	1.11	0.335	0.03
	P × C	3509.16	8	4.04	<0.001	0.45
	N × C	101,819.65	4	234.34	<0.001	13.03
	P × N × C	1445.11	8	1.66	0.126	0.18
	Residual (error)	6517.32	60			0.83
CFE_PCF_ (%)	Pattern (P)	206.25	2	3.75	0.029	1.00
	Rod Number (N)	3577.94	1	130.19	<0.001	17.41
	Configuration (C)	12,172.20	4	110.73	<0.001	59.23
	P × N	200.63	2	3.65	0.032	0.98
	P × C	140.93	8	0.64	0.740	0.69
	N × C	2383.62	4	21.68	<0.001	11.60
	P × N × C	219.23	8	1.00	0.448	1.07
	Residual (error)	1648.92	60			8.02

## Data Availability

The raw data supporting the conclusions of this article will be made available by the author on request.

## Nomenclature

Abbreviation	Definition	Unit
ANN	Artificial neural network	-
BCC	Body-centered cubic	-
C	Rod-length configuration in specimen coding	-
CFE	Crushing force efficiency	%
CFE_IPCF_	Crushing force efficiency based on initial peak crushing force	%
CFE_PCF_	Crushing force efficiency based on peak crushing force	%
CFRP	Carbon fiber-reinforced polymer	-
DLP	Digital light processing	-
EA	Energy absorption	J
F(s)	Crushing force as a function of displacement	N
FCC	Face-centered cubic	-
G	Graded rod-length configuration containing short, medium, and long CFRP rods	-
GPR	Gaussian process regression	-
IPA	Isopropyl alcohol	-
IPCF	Initial peak crushing force	N
KC-E	Empty Kelvin-cell lattice	-
L	Long CFRP rod	mm
m	Specimen mass	g
M	Medium CFRP rod	mm
MAE	Mean absolute error	-
MCF	Mean crushing force	N
MSLA	Masked stereolithography	-
N	Number of CFRP rods in specimen coding	-
N5	Specimen group reinforced with 5 CFRP rods	-
N13	Specimen group reinforced with 13 CFRP rods	-
P	CFRP rod placement pattern in specimen coding	-
P1	Core-dominated CFRP rod placement pattern	-
P2	Frame-type CFRP rod placement pattern	-
P3	Distributed CFRP rod placement pattern	-
PCF	Peak crushing force	N
RMSE	Root mean square error	-
RT	Regression tree	-
S	Short CFRP rod	mm
s	Deformation displacement	mm
SEA	Specific energy absorption	J/g
SVM	Support vector machine	-
U30	Uniform rod-length configuration with 30 mm CFRP rods	-
U40	Uniform rod-length configuration with 40 mm CFRP rods	-
U50	Uniform rod-length configuration with 50 mm CFRP rods	-
50/50	Hybrid rod-length configuration containing short and long CFRP rods	-
